# Therapeutic advantage of pro-electrophilic drugs to activate the Nrf2/ARE pathway in Alzheimer's disease models

**DOI:** 10.1038/cddis.2016.389

**Published:** 2016-12-01

**Authors:** Stuart A Lipton, Tayebeh Rezaie, Anthony Nutter, Kevin M Lopez, James Parker, Kunio Kosaka, Takumi Satoh, Scott R McKercher, Eliezer Masliah, Nobuki Nakanishi

**Affiliations:** 1 Neurodegenerative Disease Center, Scintillon Institute, San Diego, CA 92121, USA; 2 Department of Neurosciences, University of California, San Diego, School of Medicine, La Jolla, CA 92093, USA; 3 Neuroscience and Aging Research Center, Sanford-Burnham-Prebys Medical Discovery Institute, La Jolla, CA 92037, USA; 4 Research and Development Center, Nagase CO., LTD., Kobe, Hyogo 651-2241, Japan

## Abstract

Alzheimer's disease (AD) is characterized by synaptic and neuronal loss, which occurs at least partially through oxidative stress induced by oligomeric amyloid-*β* (A*β*)-peptide. Carnosic acid (CA), a chemical found in rosemary and sage, is a pro-electrophilic compound that is converted to its active form by oxidative stress. The active form stimulates the Keap1/Nrf2 transcriptional pathway and thus production of phase 2 antioxidant enzymes. We used both *in vitro* and *in vivo* models. For *in vitro* studies, we evaluated protective effects of CA on primary neurons exposed to oligomeric A*β*. For *in vivo* studies, we used two transgenic mouse models of AD, human amyloid precursor protein (hAPP)-J20 mice and triple transgenic (3xTg AD) mice. We treated these mice trans-nasally with CA twice weekly for 3 months. Subsequently, we performed neurobehavioral tests and quantitative immunohistochemistry to assess effects on AD-related phenotypes, including learning and memory, and synaptic damage. *In vitro*, CA reduced dendritic spine loss in rat neurons exposed to oligomeric A*β*. *In vivo*, CA treatment of hAPP-J20 mice improved learning and memory in the Morris water maze test. Histologically, CA increased dendritic and synaptic markers, and decreased astrogliosis, A*β* plaque number, and phospho-tau staining in the hippocampus. We conclude that CA exhibits therapeutic benefits in rodent AD models and since the FDA has placed CA on the ‘generally regarded as safe' (GRAS) list, thus obviating the need for safety studies, human clinical trials will be greatly expedited.

Alzheimer's disease (AD) affects 5% of people over age 65 years, and its prevalence is increasing.^1^ Although AD manifests amyloid plaques and tau tangles, loss of synapses, eventually accompanied by neuronal loss, more closely correlates with cognitive decline.^2^ Damage to neurons occurs at least partially through generation of oxidative and nitrosative stress, due to excessive generation of reactive oxygen/nitrogen species (ROS/RNS) triggered by oligomeric amyloid-*β* (A*β*) peptide.^3, 4, 5, 6^

Activation of the Keap1/Nrf2 (kelch-like ECH-associated protein 1/nuclear factor erythroid 2-related factor 2) pathway upregulates transcription of phase 2 antioxidant and anti-inflammatory proteins. We and others have shown that this can be a valuable therapeutic strategy in several neurodegenerative diseases (reviewed in Satoh *et al.*^7^). Here, we tested this approach in mouse models of AD using a compound known to activate the Keap1/Nrf2 pathway and to be clinically tolerated because of its presence in herbs widely-used in cooking. A critical factor that suggested the use of Nrf2 activators in this context was the finding that deficits in spatial learning, as seen in amyloid precursor protein (APP) transgenic mice, were ameliorated after intra-hippocampal injection of a lentiviral vector expressing Nrf2.^8^ Conversely, in humans with AD, decreased expression of Nrf2 in hippocampal neurons and astrocytes has been reported.^9^ The Keap1/Nrf2 pathway can be activated by an electrophilic compound when it reacts with a specific thiol on Keap1, releasing Nrf2 in the cytoplasm so it can enter the nucleus, where it binds to the antioxidant response element (ARE) on the promoters of phase 2 genes.^10^ Interestingly, several exogenous electrophilic compounds, including natural products, have been shown to activate this pathway, and thereby provide neuroprotection against ROS/RNS.

An important issue, however, with electrophilic drugs is that they non-specifically react with thiol groups, and because glutathione (GSH) is the predominant thiol in normal cells, GSH can be depleted by electrophiles thus paradoxically lowering the threshold for cell toxicity in unstressed cells.^11^ An alternative strategy is to use pro-electrophilic drugs (PEDs) that are activated by the very oxidation in redox-stressed cells that is injurious and in which GSH has already been depleted.^7^ Carnosic acid (CA) represents such a PED that is found in the herbs rosemary and sage, which reportedly exhibit antioxidant and anti-inflammatory properties.^12, 13^ Our group elucidated the mechanistic basis for this action, showing that CA is relatively innocuous until activated by ROS at the site of damage. We demonstrated that ROS converts CA to the active electrophilic form, which affords protection in animal models of neural damage^7, 14^

In the present study, we demonstrate the neuroprotective effects of CA both *in vitro* and *in vivo* in two transgenic mouse models of AD. Our neurobehavioral and histological readouts suggest that CA, administered trans-nasally *in vivo*, can be an effective treatment for AD in these rodent models.

## Results

### CA treatment ameliorates A*β*-induced spine loss in cultured rat cortical neurons

**Figure 1:**
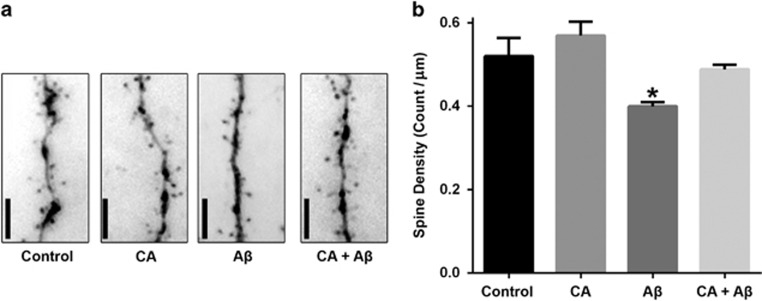
Carnosic acid (CA) treatment ameliorates A*β*-induced dendritic spine loss in cultured rat neurons. (**a**) Images of rat cortical neurons transfected with pmax-GFP and then exposed to synthetic A*β*_42_ (250 nM oligomers) with or without CA (10 *μ*M) for 4 days. After fixation, dendritic spines were visualized by GFP fluorescence. (**b**) Quantification of spine density per micrometer of dendritic length. Neurons exposed to oligomeric A*β* manifested significantly reduced dendritic spine density, and treatment with CA ameliorated this loss. Values are mean+S.E.M. (*n*>20 fields of dendritic spines for each condition; *P<0.05 by ANOVA compared with other conditions). Scale bar, 5 *μ*m

We first assessed potential therapeutic benefits of CA in primary cortical neurons prepared from rat embryos. After 14–16 days in culture, neuronal cells were transfected with pmax-GFP to visualize dendritic spines. We then exposed the transfected cells to synthetic amyloid-*β* peptide 1-42 (A*β*_42)_ oligomers (250 nM) in the presence or absence of CA. Four days after exposure, we fixed the neurons and quantified the number of dendritic spines per micrometer of dendritic length. As shown in Figure 1, A*β* exposure significantly reduced dendritic spine density in rat cortical neurons compared to control. In contrast, CA treatment concomitant with the A*β* exposure ameliorated oligomeric A*β*-induced dendritic spine loss.

### CA treatment rescues deficits in spatial learning in hAPP-J20 AD mice

**Figure 2:**
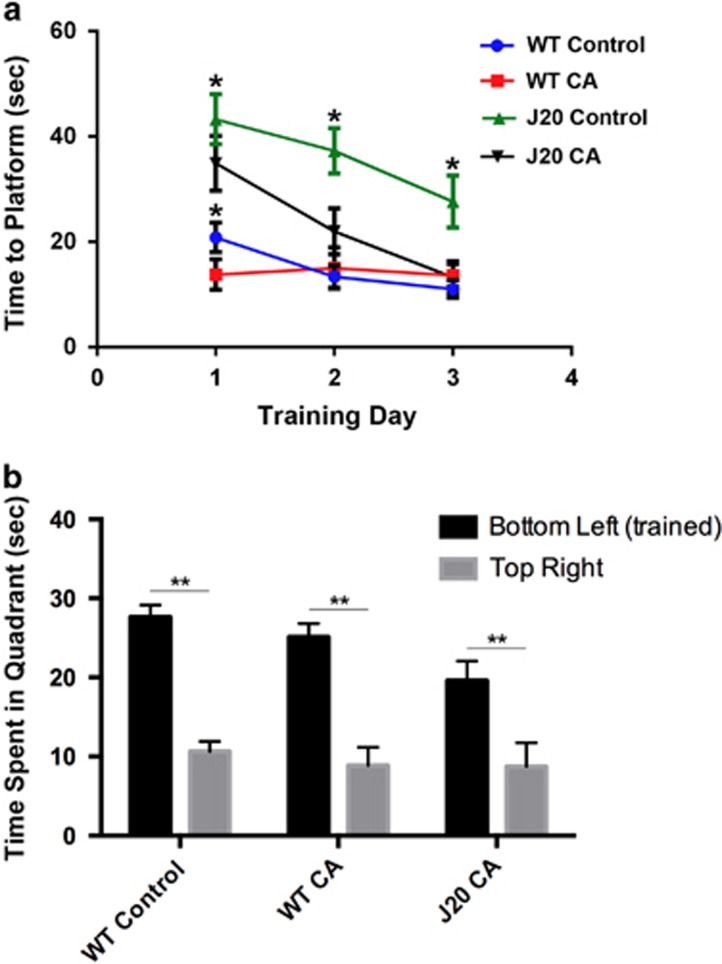
CA treatment improves spatial learning in hAPP-J20 mice. (**a**) J20 and WT littermate controls were assessed for learning in the Morris water maze test after treatment with CA or vehicle (Control). Evaluation of time to find the hidden platform during training in the four groups of mice indicates that CA treatment improved spatial learning in J20 mice (*n*⩾6 mice/group; **P<0.03 by two-way ANOVA with Tukey's multiple comparisons test). (**b**) Following training, a probe test, in which the hidden platform was removed, was performed to assess spatial memory. Mice were scored for the time spent in the bottom left quadrant (prior location of the platform) versus the top right quadrant (*n*⩾5 mice/group; **P<0.01 by Student's *t*-test). The performance of J20-CA mice is not significantly different from those of WT-Control and WT-CA mice by two-way ANOVA with Tukey's multiple comparisons test. Dashed line indicates time spent in a quadrant by chance alone (15 s)

We assessed the effects of treatment with CA on spatial learning in hAPP-J20 mice. To this end, both wild-type (WT) and hAPP-J20 mice received transnasal CA administration for 3 months beginning at the age of 3 months. We then evaluated these mice in the Morris water maze. As shown in Figure 2a, after acclimatization and during the hidden platform trials, we found that on all three days (training days 1-3) hAPP-J20 mice without CA treatment spent significantly longer times in reaching the hidden platform than WT mice (treated with either vehicle or CA (WT-Control and WT-CA)). These data indicate that hAPP-J20 mice exhibited substantial deficits in the learning phase of the water maze test. This finding is consistent with previous reports that showed learning deficits in 6-month-old hAPP-J20 mice.^15^ Importantly, however, although the hAPP-J20 mice that received CA treatment (J20-CA) took a significantly longer time to reach the platform on the first day of training compared to WT, by subsequent training day 3 they took a similar amount of time to reach the platform (Figure 2a). This finding indicates that treatment with CA improves learning in hAPP-J20 mice. We next tested memory function in these mice. However, because we found that only a small number of hAPP-J20 vehicle-treated (control) mice learned the location of the hidden platform during our training protocol (Figure 2a), their memory could not be tested. Therefore, we performed a ‘non-inferiority trial' to compare the effect of CA on hAPP-J20 mice vs. WT to determine if they were statistically different or not. We tested three groups of mice (WT-Control, WT-CA and J20-CA) in probe trials in which the platform was removed from the pool and the time spent in different quadrants of the pool was recorded during a 60-second-long trial. As shown in Figure 2b, all three groups of mice spent significantly more time in the trained quadrant (bottom left) than in the opposite quadrant (top right*).* These data are consistent with the notion that these mice remembered the location of the hidden platform following the three days of training. Furthermore, we found that the performance of the J20-CA mice was not significantly different from the WT-Control and WT-CA groups. Collectively, these results indicate that treatment with CA improved hAPP-J20 mice in the learning phase of the Morris water maze test and normalized their performance in the memory phase.

### CA Treatment rescues dendritic loss in hAPP-J20 mouse brain, as reflected by the number of MAP2-positive cells

**Figure 3:**
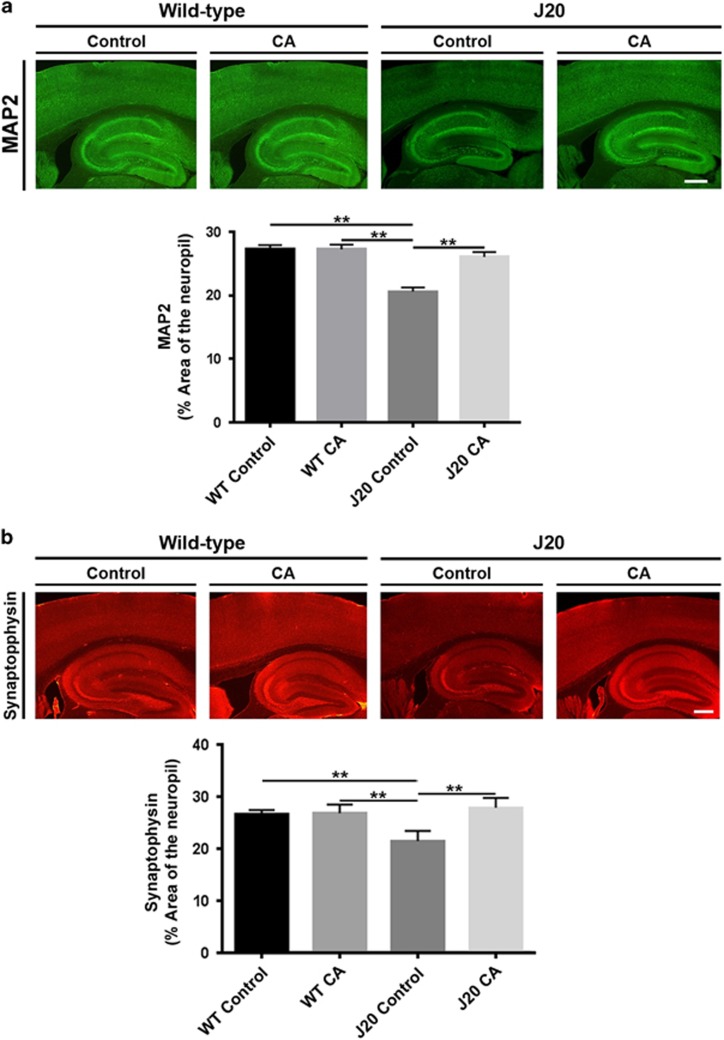
CA treatment reverses deficits in neuropil and synaptic density in hAPP-J20 mice. Quantitative immunohistochemistry of hippocampal sections prepared from wild-type (WT) and J20 mice treated with CA or vehicle (Control). (**a**) Immunohistochemistry with MAP2 antibody. CA treatment restored MAP2 staining/neuropil density to normal levels in the hippocampus of hAPP-J20 mice. (**b**) Immunohistochemistry with synaptophysin antibody. CA treatment restored synaptophysin staining/synapse density to normal levels in both cortex and hippocampus of hAPP-J20 mice (*n*=4–10 mice/group; **P<0.01 by ANOVA). Scale bar, 250 *μ*m

We next evaluated the effects of CA treatment on the dendritic/neuronal loss that occurs in hAPP-J20 mice.^16^ For this experiment, WT and hAPP-J20 mice received either vehicle or CA trans-nasally for 3 months starting at 3 months of age. We then prepared hippocampal sections from the four groups of mice (WT-Control, WT-CA, J20-Control and J20-CA) and performed quantitative confocal immunohistochemistry using MAP2 monoclonal antibody. WT-Control and WT-CA brains showed robust MAP2 signal in the hippocampus, while J20-Control exhibited a decrement in MAP2 signal (Figure 3a, top). When quantified by measuring percent (%) area of MAP2-positive neuropil (Figure 3a, bottom), J20-Control hippocampus exhibited a significant decrease in MAP2 signal. Remarkably, J20-CA hippocampus showed significantly greater MAP2 signal than that of J20-Control and similar to that of WT-Control and WT-CA. These results indicate that CA treatment can rescue the dendritic loss that occurs in hAPP-J20 mice.

### CA Treatment rescues synaptic loss in hAPP-J20 mouse brain, as reflected by synaptophysin staining

We also examined the effects of CA treatment on the synaptic loss that occurs in the hAPP-J20 mouse model of AD.^17^ Quantitative confocal immunohistochemistry, expressed as percent (%) area decorated by synaptophysin antibody, revealed a significant reduction in synaptophysin signal in the J20-Control hippocampus compared to WT (Figure 3b). In contrast, the synaptophysin immunosignal was normal in J20-CA hippocampi, indicating that CA treatment rescues synaptic loss in the hAPP-J20 hippocampus.

### CA Treatment decreases astrocytosis in hAPP-J20 mouse brain, as reflected by the number of GFAP-positive cells

**Figure 4:**
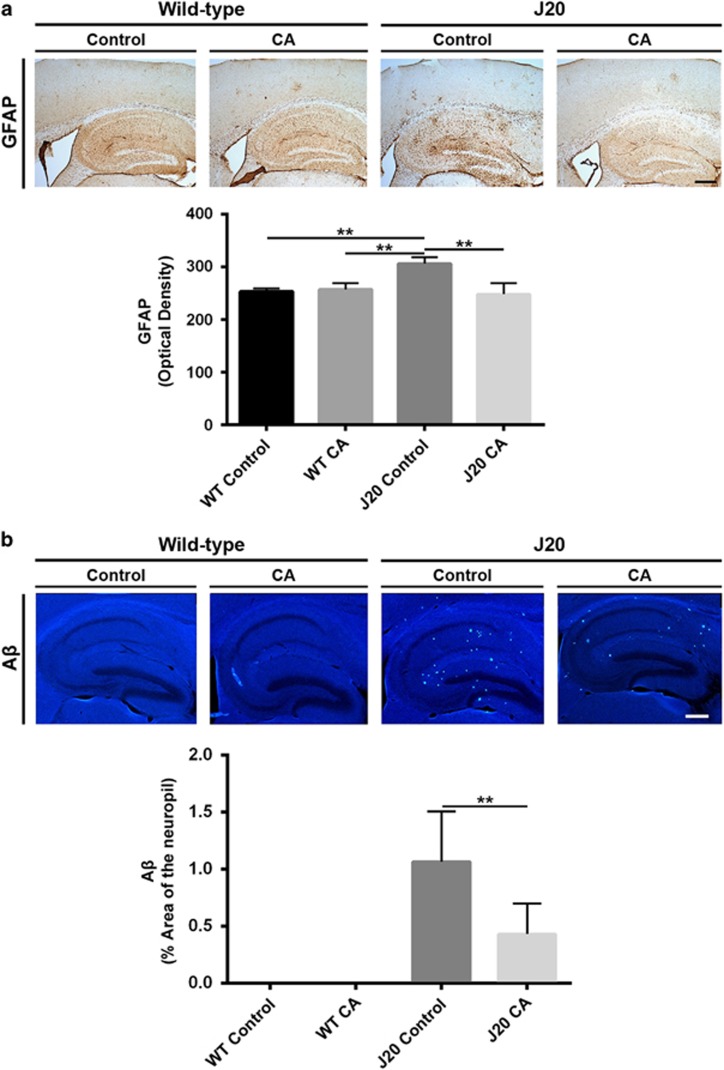
CA treatment prevents reactive astrocytosis and blocks accumulation of A*β* protein aggregates in hAPP-J20 mice. Quantitative immunohistochemistry of hippocampal sections from wild-type (WT) and J20 mice treated with CA or vehicle (Control). (**a**) Immunohistochemistry with GFAP antibody. CA treatment decreased GFAP staining/astrocytosis in the hippocampus of hAPP-J20 mice. (**b**) Immunohistochemistry with A*β* antibody. A*β* protein aggregates were significantly decreased after treatment with CA (*n*=4–10 mice/group; **P<0.01 by ANOVA). Scale bar, 250 *μ*m

We next examined the effects of CA treatment on the gliosis that occurs in hAPP-J20 mice.^17^ Immunohistochemistry using GFAP monoclonal antibody revealed a significant increase in GFAP signal in J20-Control but not in J20-CA hippocampus (Figure 4a). This finding indicates that treatment with CA can prevent gliosis in the hippocampus of hAPP-J20 mice.

### CA Treatment reduces amyloid accumulation in hAPP-J20 mouse brain, as reflected by A*β* immunohistochemistry

Previous work had shown that CA could suppress production of A*β* *in vitro,*^18^ but heretofore this has not been demonstrated in intact brain. hAPP-J20 mice express high levels of A*β* peptide in multiple brain areas, including cortex and hippocampus.^17^ Therefore, we examined the effects of treatment with CA on A*β* levels in hAPP-J20 mouse brain. Quantitative confocal immunohistochemistry was performed using anti-human A*β* 4G8 antibody (recognizing amino-acid residues 17-24 of the A*β* peptide). WT-Control treated and WT-CA treated hippocampus showed undetectable levels of A*β*. In contrast, J20-Control treated hippocampus showed high levels of A*β* signal. Remarkably, levels of A*β* were significantly reduced in J20-CA treated brain sections (Figure 4b), indicating that CA treatment suppressed A*β* levels in hAPP-J20 mice *in vivo*.

### Effects of CA treatment on 3xTg AD mouse brain

**Figure 5:**
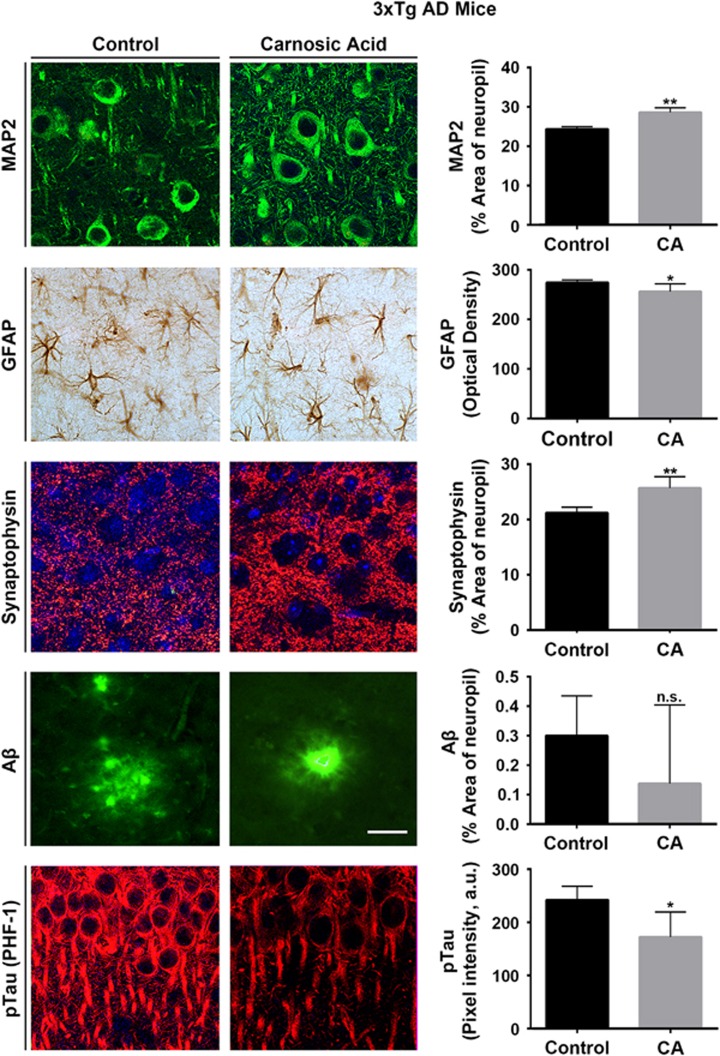
CA treatment improves AD brain markers in 3xTg AD mice. Quantitative immunohistochemistry of hippocampal sections from 3xTg AD mice treated with CA or vehicle (Control). After CA treatment, 3xTg AD hippocampus displayed increased MAP2/neuropil and synaptophysin/synaptic densities, and reduced GFAP/astrocytosis and p-tau (*n*=8–9 mice/group; *P<0.05, **P<0.01 by *t*-test). Scale bar, 40 *μ*m

Next, we assessed the effects of CA treatment in a second AD mouse model in order to test for generalization of our findings. Since these mice develop neuropathological phenotypes more slowly than hAPP-J20 mice, we started CA treatment at age 6 months of age, trans-nasally administering CA or vehicle for the next 3 months. At 9 months of age, mice were killed, and hippocampal sections prepared for immunohistochemical analysis by quantitative confocal microscopy. As shown in Figure 5, CA treatment of 3xTg mice resulted in (1) increased MAP2 signal, (2) decreased GFAP signal and (3) increased synaptophysin labeling. In addition, 3xTg mice have been reported to manifest increased amounts of p-tau.^19^ We found that CA treatment reduced the levels of p-tau signal in 3xTg mouse brain. Taken together, these data support the notion that CA treatment rescues or ameliorates pathophysiological phenotypes of AD in two separate mouse models of the disease.

## Discussion

**Figure 6:**
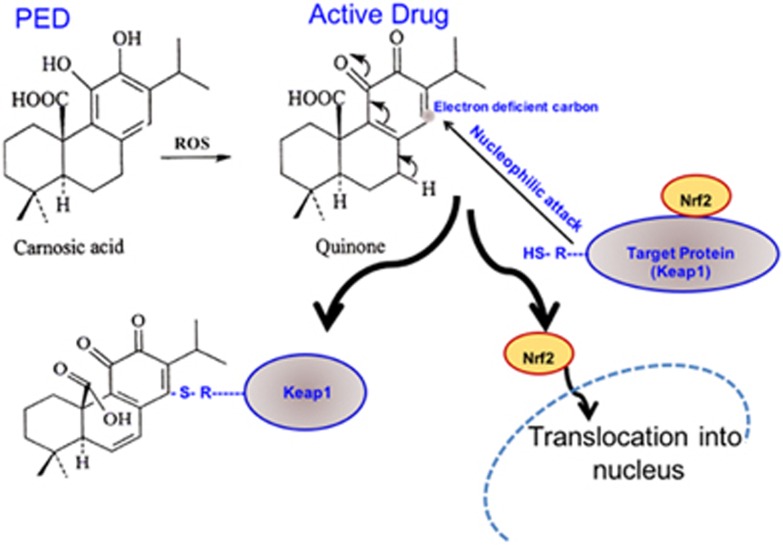
Schematic model showing action of a pro-electrophilic drugs (PEDs) in activating the Nrf2 transcriptional pathway. In this case the PED Carnosic Acid (CA) is activated by reactive oxygen species (ROS) to the active quinone form. This activated form of the drug reacts with a critical thiol (-SH) group on the cytoplasmic protein Keap1, which releases the transcription factor Nrf2. Nrf2 then is free to enter the nucleus where it transcriptionally activates a series of endogenous, neuroprotective antioxidant and anti-inflammatory proteins known as phase 2 enzymes

We have previously shown that pro-electrophilic drugs (PEDs) that activate the Keap1/Nrf2 transcriptional pathway can increase the levels of phase 2 antioxidant and anti-inflammatory enzymes in neural tissue to afford neuroprotection (Figure 6).^7, 14^ PEDs display an advantage over conventional electrophilic compounds that activate the Nrf2 pathway, including dimethylfumarate, in that they do not deplete glutathione from normal cells in the body; therefore, PEDs are expected to be better tolerated clinically than electrophilic drugs.^7^ Moreover, we and others have previously shown that CA crosses the blood-brain barrier and is detectable at neuroprotective concentrations in various CNS tissues when it is administered via various routes (intraperitoneal, oral and transnasal).^20, 21, 22^ Importantly, we have previously demonstrated ‘target engagement' of CA via its reaction with a critical cysteine residue in Keap1 to activate Nrf2 in our models. We showed activation of the Nrf2 pathway via reporter gene assay, chromatin immunoprecipitation (ChiP), electrophoretic mobility shift assay (EMSA) and RNA and immunoblot readouts of phase 2 enzymes (reviewed in Satoh *et al.*^7^).

While electrophilic compounds, including kavalactone, decursin, sulforaphane, oleanolic acid, CDDO-methylamide and curcumin, have been shown to attenuate A*β* toxicity in cell-based or animal models,^23, 24^ PEDs have not previously been tested *in vivo* in transgenic models of AD. Accordingly, in the present study, we sought to test the effects of the PED, CA, in two different transgenic mouse models of AD. Specifically, we show that CA treatment of hAPP-J20 mice for three months ameliorates impairment in learning on the Morris water maze test. We also demonstrate that CA rescues or ameliorates histological damage in the hippocampus of both the hAPP-J20 and 3xTg AD mouse models. We found that treatment with CA decreased (i) dendritic loss, assessed by MAP2 staining, (ii) synaptic loss, assessed by synaptophysin staining, (iii) gliosis, assessed by GFAP staining and (iv) deposition of A*β* peptide. In addition, in the 3xTg model in which mutant tau is expressed, CA prevented accumulation of p-tau. Collectively, these data show that treatment with CA rescues various AD-related phenotypes in hAPP-J20 and 3xTg mice.

Concerning the mechanism of these protective effects, our group previously showed that oxidative stress converts CA into an active electrophile that reacts with Keap1 at a critical cysteine residue. This results in less ubiquitination and degradation of Nrf2, leaving free Nrf2 to enter the nucleus to activate expression of endogenous phase 2 genes.^7^ These genes encode antioxidant enzymes, including heme oxygenase I (HO-1), *γ*-glutamyl cysteine synthetase (γ-GCS), NAD(P)H:quinone oxido-reductase (NQO-1) and sulfiredoxin (SRXN1).^10^ In AD, aberrant production of ROS and RNS are thought to interfere with normal cellular processes and thus contribute to the pathogenesis of neurodegeneration.^25^ Thus, it is likely that the neuroprotective effect of CA *in vivo* in AD transgenic mice occurs, at least in part, by activation of the Keap1/Nrf2 pathway and hence induction of antioxidant phase 2 enzymes.

Moreover, CA may exert other actions in neurons via activation of Nrf2 in addition to induction of antioxidant enzymes. For example, CA was recently reported to suppress A*β*_42_ production and oligomerization in the SH-SY5Y neural cell line.^18^ The authors attributed this suppression of A*β* production to transcriptional upregulation of *α*-secretase (but not *β*-secretase) by CA. *α*-Secretase induces alternate cleavage of APP, avoiding generation of A*β*_42_. The upregulation of *α*-secretase transcription is potentially mediated by Nrf2 activation, but CA has been shown to activate other transcriptional pathways as well.^7^ In addition, activation of Nrf2 has been shown to decrease the level of phospho-tau protein,^26^ which is thought to mediate neuronal damage in AD downstream of aberrant A*β* activity.^27^

In summary, based on these findings *in vitro* and in two AD transgenic mice *in vivo*, our study shows that CA represents a promising small molecule therapeutic that may be useful in AD. Furthermore, CA is found in the herbs rosemary and sage, and hence because of its long safety record is registered on the FDA's ‘generally regarded as safe' (GRAS) list. Hence, its excellent safety profile should expedite human clinical trials with this compound not only for AD, but also for other neurodegenerative diseases where oxidative stress is thought to contribute to pathogenesis.

## Materials and methods

### A*β* peptide preparations

Synthetic A*β*_42_ was suspended to an initial concentration of 1 mM in hexafluoro-isopropanol, incubated for 2 h at room temperature, and solvent was evaporated in a SpeedVac. Peptide was resuspended in DMSO to a final stock concentration of 5 mM and was kept frozen at −80 °C until use. For oligomerization, the stock solution was diluted 10-fold in MEM (Gibco, Carlsbad, CA, USA) and was then incubated at 4 °C for ⩾24 h. Oligomerized A*β* peptide preparations were sonicated immediately before use. The total concentration of A*β*_42_ was monitored by ELISA after centrifugation at 11 000 × *g* for 2 min. Western blot and light-scattering analyses of the preparations were performed before and after centrifugation to verify their oligomeric status. By dynamic light scattering, the A*β*_42_ preparation used here contained 250 nM of oligomers. Previously, we had reported in our preparations that nanomolar concentrations of synthetic A*β*_42_ oligomers or picomolar concentrations of natural A*β*_42_ oligomers prepared from human AD brain produced similar effects.^28^

### Quantification of dendritic spine density in cultured rat primary neurons

Primary cortical cultures, containing cerebrocortical neurons, some hippocampal neurons and glia, were prepared from embryonic day 16 rat pups. The cultures were transfected at 14–16 days *in vitro* with pmax-green fluorescence protein (GFP) for subsequent visualization of synaptic dendritic spines and incubated in 10 *μ*M CA or vehicle. The next day, neurons were exposed to A*β* oligomers with CA or vehicle for 4 d. Cultures were then fixed in 4% paraformaldehyde for 10 min. Images of GFP-expressing cells were acquired by deconvolution microscopy equipped with SlideBook 5.5 software (Intelligent Imaging Innovations, Inc., Denver, CO, USA). For GFP-expressing neurons, four distinct fields of secondary or tertiary dendrites were randomly selected and dendritic spine protuberances were counted in a masked fashion.

### AD Transgenic mice

All animal procedures were approved by the IACUC at Sanford-Burnham-Prebys Medical Discovery Institute. hAPP-J20 mice express a transgene encoding pathogenic human APP (with Swedish and Indiana mutations), producing a high-level of A*β*_42_ peptide.^15, 17^ 3xTg mice express transgenes encoding three familial mutations of AD in APP, presenilin 1 and tau, namely APP Swedish, MAPT P301L and PSEN1 M146V.^19^ Homozygous 3xTg AD breeders were obtained from Dr. Frank LaFerla (UC Irvine) and age-matched C57B6 control mice from Jackson Labs. Heterozygous B6.Cg-Tg(PDGFB-APPSwInd)20Lms/2 J (hAPP-J20) breeders were obtained from Dr. Lennart Mucke (Gladstone Institute/UCSF).^17^ Genotyping was performed by PCR.

### CA treatment

To minimize systemic delivery and maximize CNS levels, CA was administered trans-nasally (10 mg/kg b.w.), as previously described.^7, 21^ We administered drug twice weekly for 3 months, commencing at age 6 months for 3xTg AD mice and age 3 months for hAPP-J20 mice (since they become symptomatic earlier). CA was dissolved in a small volume of DMSO as a stock solution, then diluted ~1000 × in a mixture of hydroxypropyl-*β*-cyclodextrin (HP-*β*-CD) and chitosan. Chitosan was used as a ‘barrier-modulating agent,' as it is known to enhance drug delivery by safe and temporary permeabilization of olfactory mucosa. As a control, we administered vehicle alone (HP-*β*-CD and chitosan). During drug delivery, animals were anesthetized with isoflurane using a nose cone. Mice were placed in a supine position and 12 *μ*l drug solution were administered by pipette in 2 μl drops, alternating between each nare. During drug administration, mice were briefly (~5 s) removed from the nose cone. Mice were then returned to the nose cone for one minute before repeating the procedure.

### Morris water maze neurobehavioral testing

The maze consisted of a circular pool filled with opaque water (21±1 °C). Distinct cues were placed around the interior of the pool above the water line. On day 0, mice were given four acclimatization trials in which they had to swim in the circular tank with a submerged platform made visible by a bright red conical beacon. Mice that did not mount the platform were guided to it gently and allowed to sit on it for 10 s before being removed by the experimenter. The maximum time allowed per trial in this task was 60 s. We placed the visible platform at the same location where the hidden platform would be later placed during the hidden platform training. We found that this protocol accelerates the learning of the platform location in WT mice. For hidden platform experiments, mice underwent training trials in which each mouse was given a maximum of sixty seconds to find the platform. If after 60 s the mouse had not located the platform, it was guided to the platform and allowed to stay on it for 10 s before being removed. The animal's movements with respect to the platform's location were recorded by an automated tracking system and analyzed.

### Brain immunohistochemistry

Brains were fixed in freshly prepared 4% paraformaldehyde (PFA) and cut into 40 μm-thick vibratome sections. Immunolabeling was performed using mouse monoclonal antibodies against neuronal-specific proteins: microtubule associated protein 2 (MAP2, 1:100; Millipore, Billerica, MA, USA), glial fibrillary associated protein (GFAP, 1:1000; Chemicon, Temecula, CA, USA), phospho-tau (p-tau, catalog #AT8, 1:500; Pierce, Waltham, MA, USA; catalog #AT270, 1:500; Pierce) and synaptophysin (1:500; Millipore). After overnight incubation with the primary antibodies, sections were incubated with Texas red or FITC-conjugated horse anti-mouse IgG secondary antibody (1:75; Vector Laboratories, Burlinggame, CA, USA), transferred to SuperFrost slides (Fisher Scientific, Waltham, MA, USA) and mounted under glass coverslips with anti-fading media (Vector). For A*β* quantification, tissues were pretreated with 90% formic acid for 1 min, rinsed and incubated in 0.6% hydrogen peroxidase in tris-buffered saline (TBS) with 5% goat serum and 0.25% Triton X-100 to block nonspecific labeling. Sections were then washed and incubated in anti-human A*β* 4G8 antibody (1:1000; Signet, Dedham, MA, USA) overnight, followed by sequential treatment with biotinylated goat anti-mouse secondary antibody (Jackson ImmunoResearch Laboratories, West Grove, PA, USA), avidin-biotin solution (ABC Elite; Vector Laboratory) and nickel-enhanced diaminobenzidine. Signals were analyzed by quantitative confocal immunofluorescence using blind-coded sections, serially imaged on a laser-scanning confocal microscope and quantified using NIH Image 1.43 software. A total of three sections for each brain and four fields for each section were analyzed in each brain area.

### Statistical assessment

Pairwise comparisons were made using a two-tailed Student's *t*-test. For multiple comparisons, an ANOVA was performed followed by an appropriate post-hoc test. A *P*-value of <0.05 was considered statistically significant.
